# The Feeding of the Sick

**Published:** 1897-02

**Authors:** 


### THE FEEDING OF THE SICK.

Whilst it must be admitted that a great deal has been, is still, and will in future continue to be said upon the subject of feeding the sick, there is one simple proposition which has not been sufficiently emphasized. This is, that it is far better to give no food at all during suspension of the digestive functions than to cram down food with the irrational hope that when it is inside the patient it is in the patient. A beefsteak or a cup of milk in an incapacitated stomach yield no nourishment, for they are neither digested or absorbed, but lie there foreign bodies in an ill organ, causing irritation and its train of sequalæ. The same beefsteak laid upon the external integument, while of no benefit to the patient, would at least not cause the same irritative disturbance.

It is safe to lay down the general rule that in acute cases of asthenic or strong type, food should not be given in any form until it is craved, even though none be administered for four or five days. Many acute gastric disturbances disappear after twenty-four hours abstention from food. Many persons over-feed and thus block their own eliminative organs with waste material quicker than it can be thrown off. Hearty feeding is notoriously prone to disincline one to any kind of exertion, thus cutting off the very best means of effecting thorough elimination.

We would not be understood as advocating a starvation plan of treating the ill and well. By no means. There are many cases that must be well fed from the beginning of their illness, because they need it. Such are acute asthenics and chronic cuses.

Probably one of the best guides to feeding, in a large proportion of cases, is the desire of the patient; but, of course, not in all. Just as an otherwise well person after long fasting will lose all appetite and have only a feeling of general weakness, so also will many ill persons lose all desire for food, however great may be their need of it. It is here that the discriminating judgment of the physician comes into play. A well-acting heart, a good pulse, is a fair index that the patient may safely go without food or with a minimum allowance, as his appetite or its absence may dictate. There is no sense in feeding a patient on the one hand whilst depletory remedies are being used to counteract the stimulating effect of food.

It has often seemed to us that the heavily coated tongue and nausea, at the inception of acute asthenic disease, was Nature's mute command to supply no more nourishment until the system required it, and had adapted itself to its change into blood. We have aborted many incipient "bilious attacks" and acute dyspepsias by total abstention from food for twenty-four hours, using, however, during this time copious draughts of water to favor elimination—to flush the body.—*Medical World.*

